# Supplementary material for: The interactome of intact mitochondria by cross-linking mass spectrometry provides evidence for coexisting respiratory supercomplexes
Source: Mol Cell Proteomics. 2017 Dec 8;17(2):216–32. doi: 10.1074/mcp.RA117.000470 (PMC5795388; doi:10.1074/mcp.RA117.000470)
Supplement: Supplemental Data [file supp_RA117.000470_133922_0_supp_23978_fzffwf.zip › spectra_annotation/mito_DR_spectra_annotation/189-1-1-1-7-1.pdf]

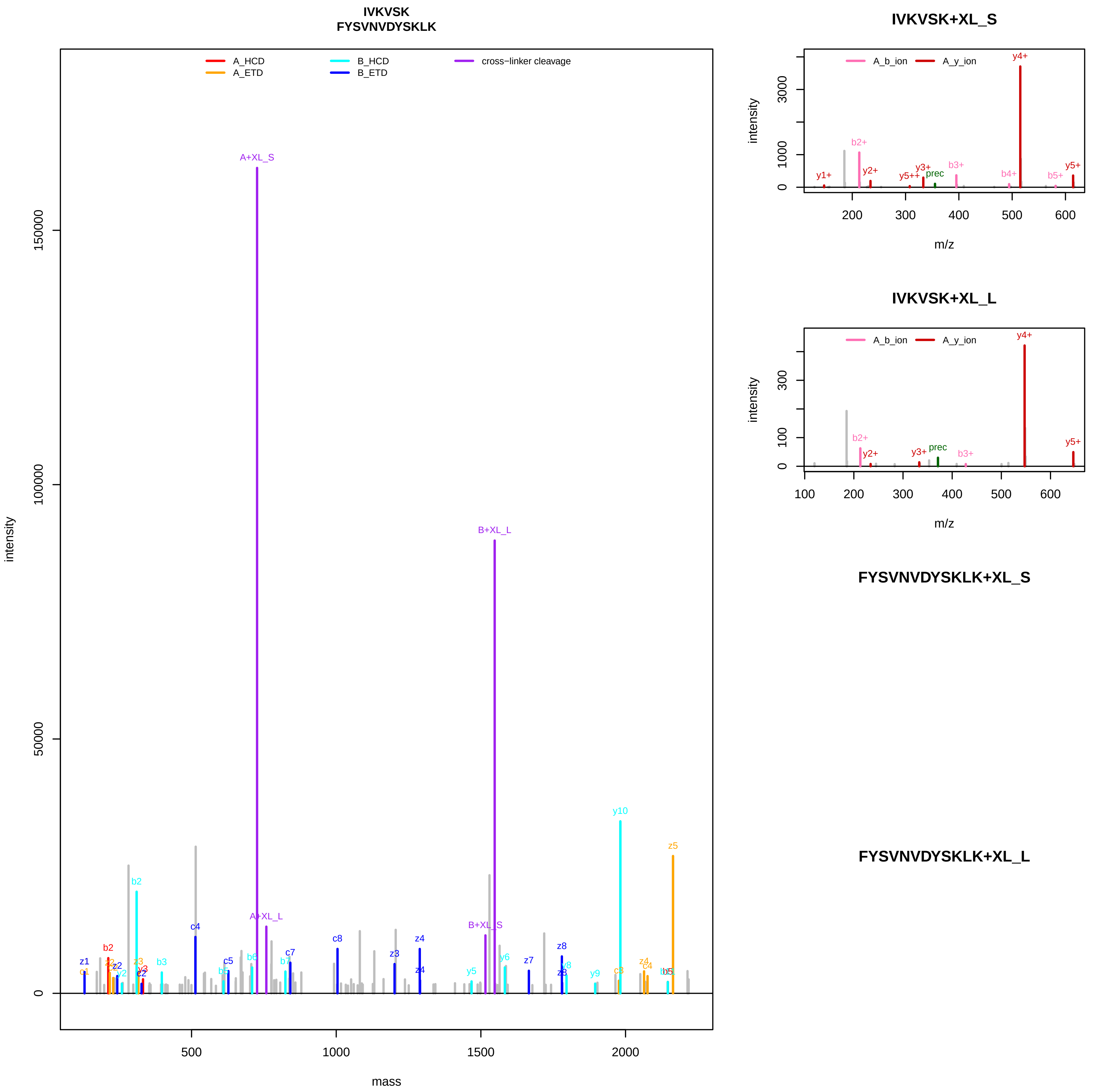

IVKVSK
FYSVNVDYSKLK
A_HCD
A_ETD
B_HCD
B_ETD
cross−linker cleavage
A+XL_S
B+XL_L
A+XL_L
B+XL_S
y10
z5
b2
c4
c8
z4
z3
z8
z7
c7
intensity
mass
0
50000
100000
150000
500
1000
1500
2000
IVKVSK+XL_S
A_b_ion
A_y_ion
y4+
b2+
y2+
y1+
y5++
y3+
prec
b3+
b4+
b5+
y5+
0
1000
3000
200
300
400
500
600
m/z
intensity
IVKVSK+XL_L
A_b_ion
A_y_ion
y4+
b2+
y2+
y3+
prec
b3+
y5+
0
100
300
100
200
300
400
500
600
m/z
intensity
FYSVNVDYSKLK+XL_S
FYSVNVDYSKLK+XL_L
